# Hand-foot-and-mouth disease virus receptor KREMEN1 binds the canyon of Coxsackie Virus A10

**DOI:** 10.1038/s41467-019-13936-2

**Published:** 2020-01-07

**Authors:** Yuguang Zhao, Daming Zhou, Tao Ni, Dimple Karia, Abhay Kotecha, Xiangxi Wang, Zihe Rao, E. Yvonne Jones, Elizabeth E. Fry, Jingshan Ren, David I. Stuart

**Affiliations:** 1 Division of Structural Biology, The Wellcome Centre for Human Genetics, University of Oxford, Headington, Oxford, OX3 7BN UK; 2 Materials and Structural Analysis, Thermo Fisher Scientific, Eindhoven, The Netherlands; 3 National Laboratory of Macromolecules, Institute of Biophysics, Chinese Academy of Science, 100101 Beijing, China; 4 Diamond Light Source Ltd, Harwell Science & Innovation Campus, Didcot, OX11 0DE UK

**Keywords:** Virus structures, Cryoelectron microscopy, Viral infection

## Abstract

Coxsackievirus A10 (CV-A10) is responsible for an escalating number of severe infections in children, but no prophylactics or therapeutics are currently available. KREMEN1 (KRM1) is the entry receptor for the largest receptor-group of hand-foot-and-mouth disease causing viruses, which includes CV-A10. We report here structures of CV-A10 mature virus alone and in complex with KRM1 as well as of the CV-A10 A-particle. The receptor spans the viral canyon with a large footprint on the virus surface. The footprint has some overlap with that seen for the neonatal Fc receptor complexed with enterovirus E6 but is larger and distinct from that of another enterovirus receptor SCARB2. Reduced occupancy of a particle-stabilising pocket factor in the complexed virus and the presence of both unbound and expanded virus particles suggests receptor binding initiates a cascade of conformational changes that produces expanded particles primed for viral uncoating.

## Abstract (web summary)

Here, the authors provide the structure of mature Coxsackie Virus A10 alone and in complex with its receptor KREMEN1, and of A-particles. This shows how the receptor spans the viral canyon and suggests that receptor binding triggers pocket factor release and conformational changes resulting in expanded particles.

## Introduction

Within the family *Picornaviridae* the genus Enterovirus is the most populous and the most important for human health. It is currently classified into 15 species of unenveloped, single-stranded, positive-sense RNA viruses^1^ responsible for a broad range of human and mammalian diseases including the common cold, hand-foot-and-mouth disease (HFMD) and poliomyelitis^2^. Each species is further classified into a number of different (sero)types, species A alone currently comprises 25 types. The icosahedral capsid contains 60 copies of a protomeric unit composed of four proteins, VP1-4. VP1-3 each fold as a β-barrel with the N-termini internal and the C-termini external. VP4 is entirely internal to the capsid. Five protomers assemble into a pentamer, 12 copies of which form the spherical capsid, with VP1 associating around the icosahedral fivefold axes, and VP2 and VP3 around the twofold and threefold. Enteroviruses are unique in harbouring a lipid molecule (pocket factor) within a pocket in the VP1 β-barrel, which lies below the surface of a deep depression encircling each fivefold axis, termed the canyon. The canyon is the engagement site for slender immunoglobulin (Ig)-like receptors, as predicted by Rossmann et al.^3^. The binding of such receptors can trigger pocket factor release and viral expansion, leading to externalization of the N-terminus of VP1 followed by VP4 to form a pore in the endo/lysosome membrane through which the genome is thought to be subsequently released^4,5^. The expanded intermediate is termed the A-particle prior to genome release and B-particle subsequent to genome release^5–8^. The expanded particles have altered antigenic properties compared with the native mature particle.

More than 20 types of enteroviruses (both species A and B) have been associated with HFMD^9,10^. Earlier outbreaks in the Asia-Pacific region were predominantly caused by EV-A71 and CV-A16 but those attributable to CV-A6 and CV-A10 have become increasingly common in recent years^11–13^. CV-A10 shares only ~69% amino acid sequence identity with EV-A71 and CV-A16, resulting in changes in the surface architecture^14^ and recognition of a different cell entry receptor. Indeed HFMD viruses can be divided into four groups depending on their receptor usage (Supplementary Fig. 1): EV-A71, CV-A7, CV-A14 and CV-A16 use SCARB2 (scavenger receptor class B member 2, also named lysosomal integral membrane protein-2, LIMP-2)^15,16^, Coxsackie viruses A2-6, A8, A10 and A12 use KREMEN1 (kringle (KR) containing transmembrane protein 1; KRM1)^17^, Coxsackie viruses B1-3 and B5 use CAR (Coxsackievirus and adenovirus receptor) and EV-E3, E6, E7, E11 and E12 use DAF/FcRn (decay-accelerating factor/neonatal Fc receptor)^2,18,19^.

KRM1 is a non-Ig-like type I transmembrane protein. It was identified as a receptor of the secreted protein Dickkopf1 (Dickkopf-related protein 1, DKK1), a negative regulator of WNT signalling, and can amplify the antagonistic effect of DKK1 by forming a ternary complex with DKK1 and the WNT co-receptor LRP6^20^. The 40 kDa ectodomain of KRM1 comprises, from N- to C-terminus, three similarly-sized structural domains: KR, WSC (cell wall stress‐responsive component) and CUB (for complement C1r/C1s, Uegf, Bmp1) domain^21^. Crystal structures of the KRM1 ectodomain in isolation, and in complex with DKK1 and LRP6, have shown that these three domains form a substantial rigid triangular structure^21^.

When the first enterovirus structures were determined it was proposed that whilst slender receptors composed of a string of single Ig-like domains would bind within the canyon, bulkier molecules (e.g., antibodies) would be blocked from penetrating the canyon, secluding receptor binding residues from immune recognition^3^. It was thought that binding inside the canyon was required to trigger the conformational changes in the capsid required for uncoating. The actual situation has turned out to be more complex, thus the bulky SCARB2 molecule binds just to the south of the canyon of EV-A71, but still manages to trigger the required conformational changes, by an alternative mechanism^22^. In contrast another relatively large receptor, neonatal Fc receptor (FcRn), binds EV-E6 at, and penetrates into, the canyon^19^. Thus, the original rules of engagement for receptor binding require revision. KRM1 provides another example of a relatively bulky virus uncoating receptor from which to gain insight, however, no receptor complex has been visualized for KRM1-binding viruses.

Here, we have determined three cryo-EM structures following mixing of CV-A10 Kowalik strain with KRM1: the CV-A10 mature virus, a disassembly intermediate A-particle and the mature virus in complex with KRM1. The KR and WSC domains of KRM1 bind across the CV-A10 canyon making largely hydrophilic interactions with surface loops constituting the north and south rims and extend along the canyon for essentially the entire icosahedral asymmetric unit. The reduced occupancy of pocket factor in the complexed-virus particles and the presence of both unbound and expanded full particles suggests that receptor binding triggers pocket factor release, initiating the cascade of conformational changes that produces expanded A-particles. These A-particles are unable to bind receptor and are ready to uncoat by releasing the genome.

## Results

### Structure determination for virus and its receptor complex

CV-A10 (Kowalik strain) grown in HeLa cells was purified by sucrose density centrifugation. Of the three bands visible on the gradient, the material used in the cryo-EM analysis was derived exclusively from the lower band. Thermofluor and cryo-EM analysis showed that these particles were mature virus rather than A-particles (“Methods” section and Supplementary Fig. 2). The extracellular domain of KRM1 was co-expressed with DKK1 to avoid aggregation. The KRM1/DKK1 complex was combined with CV-A10 with a molar ratio of receptor to virus of ~30:1 on an ultrathin carbon grid at room temperature and immediately vitrified (see “Methods” section). Thus, if all receptor molecules were to bind to the virus then approximately half of the receptor binding sites would be occupied. This sub-stoichiometric ratio of the receptor was chosen since at higher molar ratios the virus particles uncoated. All cryo-EM data were collected from a single grid (“Methods” section). Of the particles identified in the images about half (~54%) had receptor bound at very low occupancy or manifested irregular shapes, these were not analyzed further. Approximately 20% of particles were in the native antigenic state showing little evidence of bound receptor whilst the remainder (~26%) were split roughly equally between those that showed receptor bound (for which localized reconstruction suggested that on average 28% of the binding sites were occupied) and those that had converted to the expanded form but still contained the viral genome (A-particles). The A-particles showed no evidence of bound KRM1.

**Fig. 1:**
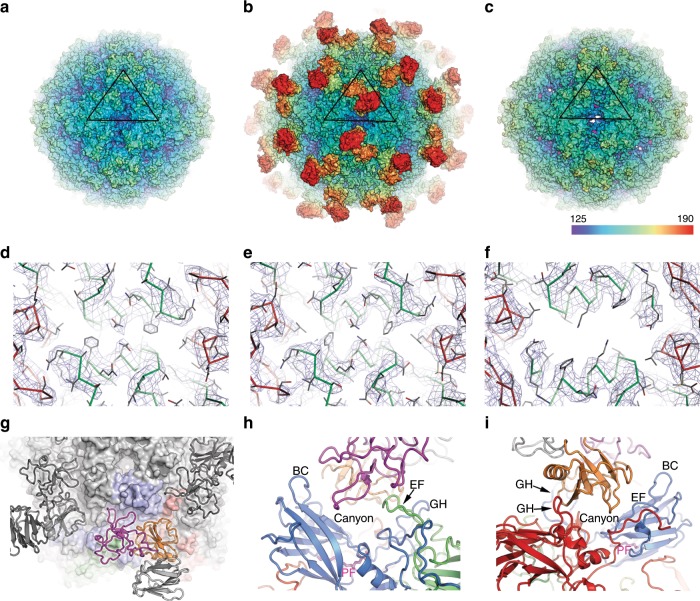
Overview structures of CV-A10 and its receptor complex. Radially coloured surface representations of mature CV-A10 virus (**a**), CV-A10/KRM1 complex (**b**) and expanded CV-A10 A-particle (**c**). The first ordered residue of the VP1 N-terminus is coloured in magenta showing where it externalizes in **c**. Electron potential maps at icosahedral twofold axis of the mature CV-A10 (**d**), CV-A10/KRM1 complex (**e**) and CV-A10 A-particle (**f**). CV-A10/KRM1 interacting across the canyon from above (**g**) where a virus pentamer is drawn as a grey surface with one icosahedral pentameric subunit coloured with VP1 blue, VP2 green and VP3 red. KRM1 is drawn as a cartoon with the domains colour coded: KR magenta, WSC orange, CUB grey, adjacent receptors are colour coded in grey. Interaction of one virus protomer with KRM1 from the side closest to the KR domain (**h**), virus and receptor drawn as a cartoon keeping the same colours as in **g**. Interaction of one virus protomer with KRM1 from the side closest to the WSC domain (**i**), drawn as for **h**.

Cryo-EM data collection, reconstruction and refinement statistics are given in Supplementary Table 1. The gold standard Fourier shell correlation curves of the final maps, maps coloured by local resolution and representative density maps of some local regions for the structures of mature virus, A-particle and receptor complex, at resolutions of 3.5, 4.4 and 3.9 Å, respectively, are shown in Fig. 1, Supplementary Figs. 3 and 4. The structure fitted to the density map of the mature CV-A10 Kowalik virion (Fig. 1a, d) comprises residues 2–297 of VP1 (residues 1 and 298 were disordered), 10–255 of VP2 (residues 1–9 were disordered), 1–240 of VP3 (no disordered residues) and 28–68 of VP4 (residues 1–27 and 69 were disordered).

### Structure of the mature CV-A10 capsid

CV-A10 Kowalik shares ~94% identity to those strains of CV-A10 for which structures are available (strain FJ-01 PDB ID: 6ACU, strain S0148b PDB ID: 6IIJ and strain SJZ10-1740T/HeB/CHN/2010 PDB ID: 6AKU)^14,23,24^, which themselves share 98% identity. As expected, the structure of CV-A10 Kowalik maintains the typical enterovirus architecture and surface topography but a signature of CV-A10 viruses is an extended VP1 BC loop that, together with the absence of helical structure in the VP1 GH loop, widens the front part of the canyon whilst the extended VP2 EF loop is bent inwards narrowing the rear of the canyon and the VP3 C-terminus protrudes into the base of the canyon. This contributes to a distinct electrostatic surface potential compared to HFMD viruses from other receptor sub-groups. In these and all other respects CV-A10 Kowalik is closely similar to previously determined CV-A10 structures^14,23,24^ (RMSDs in Cαs for superimposed polypeptides is 0.6–0.8 Å). Differences between strains are confined to the flexible surface loops suggestive of antigenic variation between them. The hydrophobic pocket within the VP1 β-barrel contains a bound aliphatic chain that has been modelled as sphingosine in accordance with other CV-A10 strains^14,23,24^. The density level for this molecule is lower than the adjacent capsid protein, suggesting an occupancy of *circa* 50% (Supplementary Fig. 5). A distinguishing feature of this strain is ordered density allowing the whole of a 13-residue long helix at the N-terminal end of VP1 to be built. This structure lies underneath the VP2 helices that abut at the twofold axes, in a similar position to, but eight residues longer than, the corresponding helix found in EV-A71 and CV-A16 (Supplementary Fig. 4c). Internally, the N-terminal portion of VP4 is disordered in CV-A10 Kowalik, but residues 18–28 are observed to cross to an adjacent biological protomer in strain SJZ10-1740T/HeB/CHN/2010^14^. Disordered RNA density is visible within the particle, the outer portion rather closely packed against the inside of the capsid (Supplementary Fig. 6).

### Structure of the CV-A10 A-particle

The CV-A10 (Kowalik) A-particle structure is similar to previously described enterovirus uncoating intermediates^14,25,26^, being ~4% expanded compared with the mature virus via a rotation of the protomeric subunit about the threefold axis and radial shifts in VP1 and VP2 relative to their adjacent icosahedral axes. It has a collapsed pocket in VP1 (due to rearrangements in the VP1 GH loop leading into strand H and the inward movement of sidechains: Met 229 and Phe 232) and no pocket factor (Supplementary Fig. 5c). The genome remains inside the particle and layers of RNA are seen inside the particle generally less closely packed against the inner surface of the capsid than in the mature particle, making the same interactions as observed previously^14,26^ (Supplementary Fig. 6). As is usual in A-particles several regions of the capsid are disordered or externalized relative to the mature virus: residues 1–65, 211–219 (GH loop) and 298 of VP1, 1–13 and 252–255 of VP2, 1, 173–182 (GH loop) and 240 of VP3 and the whole of VP4. Conformational changes relative to the mature capsid are observed in the residues preceding strand B of VP2 which in the A-particle adopt a helical structure, whilst the VP3 GH loop becomes disordered. Together these conformational rearrangements: the loss of the structure of the N-terminus of VP1 and disorder of the VP1 and VP3 GH loops, lead to openings at the icosahedral twofold axis and at the adjacent off-axis interface between VP2 and VP3^6,26^. The previously internal N-terminus of VP1 now traverses the capsid from the region of RNA interaction, via the “off-axis” channel becoming disordered or cleaved beyond the last visible residue, 66 (Supplementary Fig. 5d). This recapitulates the externalization of VP1 visualized in Poliovirus and CV-A16^25,26^ rendering the inter-pentamer interface less stable and compromising capsid integrity.

### Structure of the CV-A10 and KRM1 complex

**Fig. 2:**
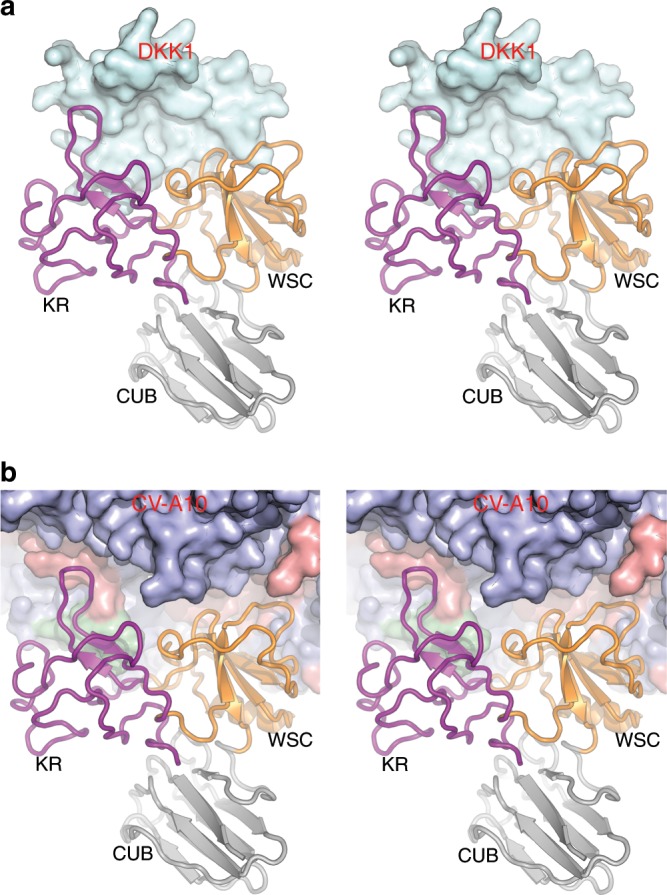
Stereo diagram showing regions of KRM1 involved in interactions with DKK1 are used in engagement with CV-A10. **a** Engagement between KRM1 and DKK1. **b** Engagement of KRM1 to CV-A10. DKK1 and CV-A10 are shown as surface representations with DKK1 in cyan and CV-A10 capsid proteins VP1, VP2 and VP3 in blue, green and red, respectively. KRM1 is drawn as ribbons with KR, WSC and CUB domains coloured in purple, orange and grey, respectively.

**Fig. 3:**
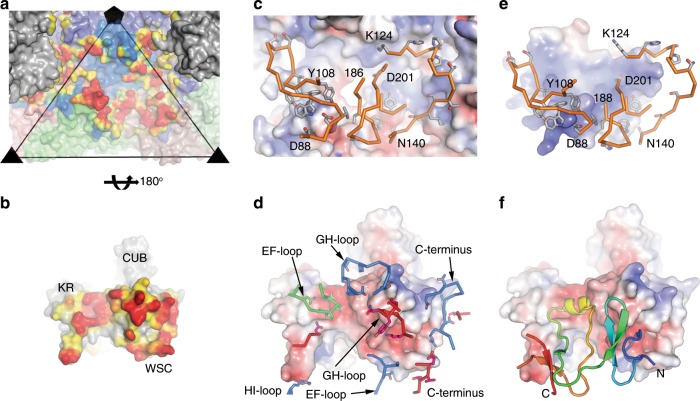
The nature of the interactions between CV-A10 and KRM1 shown as open-book views. CV-A10 (**a**) and KRM1 (**b**) interface. Both virus and receptor are shown as surface representations. VP1, VP2 and VP3 of CV-A10 are coloured in pale blue, green and red, respectively and an icosahedral subunit outlined with the fivefold (pentamer) and threefold (triangle) symmetry axes labelled, KRM1 from neighbouring subunits are shown in grey with domain names labelled in **b**. Contact areas between the virus and the receptor with distances ≤4.0 Å are shown in bright red, >4.0 Å and ≤9.0 Å in yellow. The contact areas attributable to two receptors bound at adjacent sites are shown in **a**. **c**, **d** Showing the contact areas on CV-A10 (**c**) and KRM1 (**d**) as electrostatic surfaces contoured at ±5 kT e^−1^ (blue, positive; red, negative) with key residues from the interacting partner shown as sticks with main-chain backbones in orange and sidechains grey in **c**; residues involved in contacts from the virus capsid VP1, VP2 and VP3 are shown as blue, green and red sticks, respectively in **d**. **e**, **f** Open-book view of electrostatic surfaces of interactions between KRM1 (**f**) and DKK1 (**e**). Residues that contact CV-A10 (sticks in **e**) are also involved in interactions with DKK1 in the KRM1-DKK1-LPR6 complex. DKK1 is shown as ribbons in **f**.

The CVA-10 particle seen in the receptor complex is in the native (mature) conformation and the model comprises the same residues. Receptor binding has minimal impact on the capsid structure; structural superposition yields an RMSD of 0.5 Å for Cα atoms between mature virus and complexed-virus protomers. However, there are minor conformational rearrangements in some contact regions, most notably the C-terminus of VP3 and the VP2 EF loop. Residues 136–150 of this loop constitute a linear neutralizing epitope capable of eliciting a strong antibody response when inserted in Hepatitis B core antigen to make a chimeric virus-like particle^27^. There is a noticeable reduction in the amount of bound pocket factor (to perhaps half the level seen in the mature virus particle) (Supplementary Fig. 5b). Despite the low occupancy of the receptor (~28%), careful refinement improved the receptor density to a local resolution of between 5 and 8 Å allowing cautious model fitting (Supplementary Fig. 3 and “Methods” section). Residues 29–322 of KRM1 were modelled and two of the four glycosylation sites (N45 and N59) have well-defined density for the first glycans. KRM1 engages with the virus using the same interface that attaches to DKK1^21^ (Figs. 2 and 3), thus binding to the virus has replaced DKK1 in a few seconds of soaking, suggesting that DKK1 has a reasonably fast off-rate and that KRM1 has a markedly higher affinity for CV-A10.

**Fig. 4:**
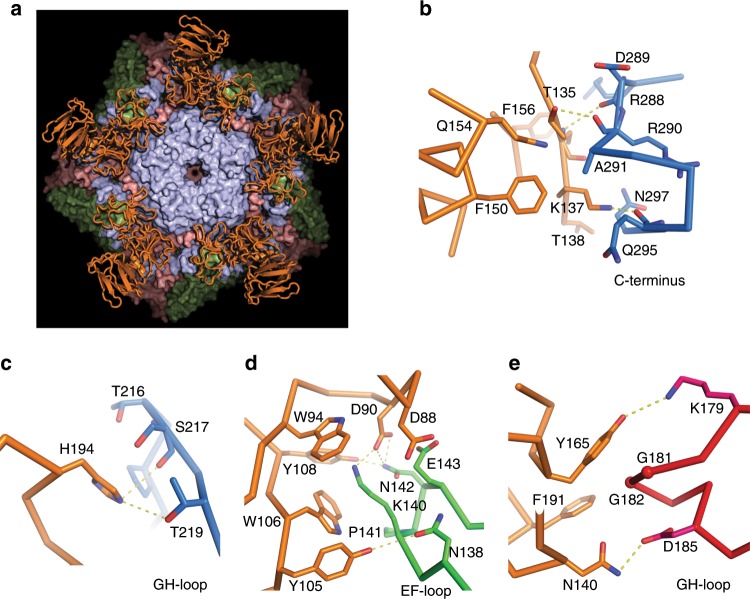
Details of interactions between CV-A10 and KRM1. **a** KRM1 (orange ribbons) engaged in the canyon around the icosahedral fivefold axis of CV-A10. Only a pentamer of the virus is shown with VP1, VP2 and VP3 as pale blue, green and red surfaces, respectively. **b**–**e** Interactions of KRM1 with VP1 C-terminus (**b**), VP1 GH loop (**c**), VP2 EF loop (**d**) and VP3 GH loop (**e**) of the virus. The main-chain backbone and sidechains of KRM1 are drawn as orange sticks, and those for CV-A10 as blue, green and red sticks for VP1, VP2 and VP3, respectively.

KRM1 binds CV-A10 with a relatively large footprint (~1400 Å^2^, Supplementary Table 2) and the first two domains of KRM1 (KR and WSC) span the virus canyon, each making interactions with both the north and south rim (Figs. 3 and 4). Although KRM1 dips into the canyon it does not reach the canyon floor (Supplementary Fig. 7). The KR domain engages at the narrow part of the canyon, contacting the C-terminus of VP3, EF loop of VP2, and the HI-loop of VP1 from a neighbouring protomer (Supplementary Fig. 8). Notably a loop spanning residues 98–102 in the KR domain, disordered in the high-resolution crystal structure, is ordered in the complex by a strong electrostatic interaction between Asp 102 at the tip of the loop with Arg 239 in the HI-loop of the adjacent CV-A10 protomer, whilst Tyr 105, Trp 106 and Tyr 108 in the same KR loop make weak interactions with Lys 140 of VP2 (Trp 106 stacking with the side chain stalk). The WSC domain of KRM1 binds the wider region of the canyon, interacting with the VP1 EF loop, VP2 EF loop, GH loop of VP1, VP3 GH loop, VP3 BC loop and the VP1 C-terminus from a neighbouring protomer (Figs. 2 and 3, Supplementary Fig. 8).

The residues involved in the virus–receptor interactions are predominately polar/hydrophilic (20 out of 26 from KRM1; 25 out of 31 from CV-A10 with distance ≤ 4.0 Å), making ten potential H-bonds and four salt bridges (Fig. 4, Supplementary Table 2). While these interactions will confer specificity on the binding much of the strength of binding likely comes from ring-stacking and hydrophobic interactions between KRM1 and the VP2 EF and VP3 GH loops, although shape complementarity is limited as the receptor does not penetrate deep into the canyon.

**Fig. 5:**
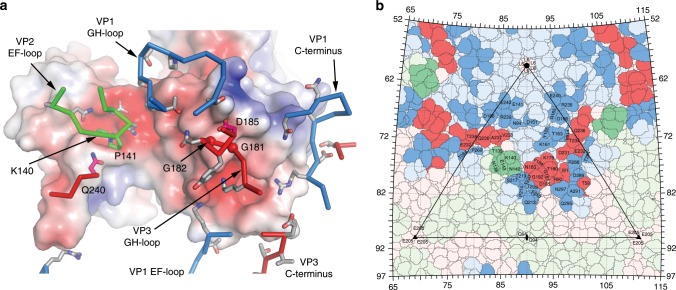
Comparison of amino acids in the receptor attachment area of CV-A10 strains and with other enteroviruses that cause HFMD. **a** Residues in 14 strains of CV-A10 are highly conserved (33 out of 37) and 6 of the 31 residues are conserved among the KRM1-dependent type A enteroviruses, these are K140 and P141 in VP2 EF loop, G181, G182 and D185 in the VP3 GH loop, and Q240 at the C-terminus of VP3. Only one residue VP3 Q240 is conserved among 19 HFDV causing enteroviruses. KRM1 is shown as an electrostatic surface, residues of CV-A10 involved in contacts are shown as sticks with the same colour scheme as Fig. 3d, except the non-conserved sidechains are in grey. **b** Roadmap depiction of the virus surface (see “Methods” section). An icosahedral subunit is outlined in black lines and residues from VP1 are shown in blue, VP2 green and VP3 red with those in contact with KRM1 highlighted in the corresponding protein colour. Residues in contact with KRM1 and around the icosahedral symmetry axis are labelled.

Receptor binding residues are highly conserved in different strains of CV-A10, but only 6 out of 31 are conserved across the 8 KRM1-dependent CVs known to cause HFMD. These are K140 and P141 in the VP2 EF loop, G181, G182 and D185 in the VP3 GH loop, and Q240 at the C-terminus of VP3 (Fig. 5 and Supplementary Fig. 8). All of these, apart from VP3 Q240 (which is also conserved across non-KRM1-binding viruses) are probably crucial for receptor binding. Capsid residues VP1 23 and 283 identified in clade E CV-A10 viruses implicated in more severe infections, do not directly interact with the receptor but residue 283 could impact on receptor binding by destabilizing the C-terminus of VP1^11^.

All enteroviruses that bind receptors in the canyon (CV-A10, PV-1, CV-A24v and E6)^19,28,29^ use the VP1 EF and GH loops, VP2 EF loop and VP3 GH loop for receptor binding (Supplementary Fig. 9). These loops are the most variable regions in sequence, length and structure among the enteroviruses.

### Two known antibodies act by blocking receptor binding

Two structures of complexes of KRM1-dependent viruses with neutralizing antibody Fabs are known, CV-A6/1D5^30^ and CV-A10/2G8^24^. 2G8 binds on a plateau south of the canyon at the VP2/VP3 boundary, interacting with the C-termini of VP1 and VP3, the EF loop of VP2 and residues preceding the B strand of VP3, whilst five 1D5 Fabs bind around the fivefold vertex, interacting with the BC, DE, EF and HI loops of VP1. Both would clash with bound KRM1, consistent with neutralization by blocking receptor binding (Supplementary Fig. 10).

## Discussion

From the analysis of enterovirus/receptor complexes, patterns are emerging. Some receptors are involved in attachment but not uncoating, e.g., LDLR and DAF that bind HRV2 and E6, respectively (Supplementary Fig. 11)^19,31^. These attachment receptors can have relatively limited areas of interaction and may bind distant from the canyon. In contrast uncoating receptors often have larger footprints (1200–1600 Å^2^) on the virus (Supplementary Table 2) and bind within the canyon (Supplementary Figs. 9 and 11). In an exception to this pattern, the uncoating receptor SCARB2 binds south of the canyon and with a footprint of only 726 Å^2^, which does not overlap that of KRM1, however, in this case, pH-induced conformation changes are thought to act in concert to help trigger uncoating^22^.

The CV-A10/KRM1 complex we report here demonstrates that relatively large non-Ig superfamily receptors can attach directly to the canyon and drive uncoating even without penetrating the canyon sufficiently to make direct interactions with the canyon floor. Receptor binding lowers the activation energy for pocket factor release and subsequent capsid conformational changes, membrane engagement and, ultimately, genome release^32^. Indeed we find that KRM1 binding to CV-A10 dislodges pocket factor. This was also observed on receptor binding to poliovirus but whereas for PV-1 the virus structure was essentially unchanged (presumably in a metastable state with a reduced activation energy)^29^, KRM1 binding to CV-A10 appears to directly trigger uncoating by conversion of particles to the altered state (A-particle)—the 10% of undecorated A-particles, we observe presumably arose through receptor catalysed conversion followed by receptor detachment, since collection from a grid with the particles incubated more briefly and less decorated with receptor did not show a significant number of A-particles, whilst incubation with higher concentrations of receptor induced uncoating. Direct evidence for receptor-mediated destablization comes from PaSTRy analysis (“Methods” section), which shows a reduction in particle stability from 55 °C to 49 °C at the highest receptor concentration (“Methods” section and Supplementary Fig. 12). The rather low occupancy of receptor (~half of the particles had no obvious decoration) is consistent with stochastic binding with a slow on-rate where high levels of binding trigger conversion. Since KRM1 binding induces very limited structural changes it is not possible to directly infer the mechanism of destablization, although despite the lack of overlap of contact regions between canyon binding receptors and the non-canyon binding SCARB2 receptor, both contact the VP2 EF loop, consistent with the mechanism proposed initially for EV-A71 being common for many enteroviruses, probably including CV-A10^6,22^. These emerging trends, commonalities and differences should help in the design of more generalized therapeutics/prophylactics.

## Methods

### Expression and purification of KRM1

Human Kremen-1 Extracellular domain (KRM1, isoform 1, residues G23-G373) was cloned into a pNeo-sec vector^33^ and a polyclonal G418 selected population of HEK293S GNTI- (ATCC CRL-3022) cells were used for secreted KRM1 production^21^. In order to avoid aggregation of the KRM1 protein, we further transfected the stable cells with the human Dickkopf-related protein 1 (DKK1, residues S141-H266) with an N-terminal fusion of a SUMO (from Saccharomyces cerevisiae), Strep-II, 8xHis tag and a Rhinovirus 3 C protease cutting site^34^. The transfected cells were incubated at 30 °C for 2 weeks and the conditioned media used for protein complex capture by nickel beads. The imidazole eluted KRM1/DKK1 protein complex was further purified by size exclusion chromatography with an S200 column (GE Healthcare).

### Production and purification of CV-A10

CV-A10 Kowalik strain (kindly provided by Professor Thijn R. Brummelkamp) was expanded in HeLa (ATCC CCL-2) cells. HeLa cells were grown in roller bottles in DMEM supplemented with 10% FBS. Before infection, the media was changed to serum free and CV-A10 (MOI of ~10) was added. Two days after infection, virus was harvested and 0.5% (v/v) NP40 was added. 8% (w/v) PEG 6000 was then added to precipitate virus. The sample was centrifuged at 3500 × *g* for 1 h at 4 °C, the resultant pellet suspended in ~35 ml buffer A (50 mM HEPES, 200 mM NaCl, 0.5% (v/v) NP40, pH 8.0) and centrifuged at 3500 × *g* for 30 min at 4 °C to remove cell debris. Virus particles in the supernatant were pelleted through a 3 ml 30% (w/v) sucrose cushion in buffer B (50 mM HEPES pH 8.0, 200 mM NaCl) at 105,000 × *g* for 3 h at 4 °C. The pellet was suspended in buffer A and centrifuged at 12,000 × *g* for 30 min at 4 °C to remove insoluble material. The supernatant was then layered on top of a 15–45% sucrose gradient (in buffer B) and centrifuged at 105,000 × *g* for 3 h at 4 °C. Fractions containing CV-A10 full particles were harvested, sucrose in the sample was removed using a desalting spin column (Zeba, Pierce) and virus particles in buffer B were concentrated using a 300 kDa ultrafiltration tube (Amicon).

### Cryo-EM sample preparation

Three microlitre of CV-A10 virus (0.2 mg/ml) was applied to a glow-discharged ultrathin carbon grid (Agar Scientific) and mixed with 0.5 µl KRM1/DKK1 protein complex (0.3 mg/ml in 20 mM Tris, 200 mM NaCl, pH 8.0) at room temperature. This was immediately blotted by filter paper and vitrified by plunging into liquid ethane using a manual cryoplunger (designed by Prof. W. Baumeister, Max Planck Institute of Biochemistry) so that vitrification occurred within a few seconds of mixing.

### Cryo-EM data collection

Data collection was carried out using a Tecnai “Polara” microscope (FEI) equipped with a Gatan GIF Quantum energy filter with an energy selecting slit width of 30 eV, operated at 300 kV. Data images were recorded on a Gatan K2 Summit direct electron detector operating in counting mode, as movies each containing 32 frames with a total electron dose of 35 e^−^ Å^−2^, using serial EM. The defocus range was 0.5–2.5 μm. The calibrated magnification was ×37,037, corresponding to a pixel size of 1.35 Å. The dose rate was ~4 e^−^ Å^−2^ s^−1^.

### Cryo-EM data processing

Frames of each movie were aligned and averaged using MotionCorr2^35^ and the contrast transfer function parameters were determined with CTFFIND3^36^. Micrographs with astigmatism or significant drift were discarded. Particles were picked using xmipp3 (with a manual initial step following by automatic picking)^37^. A total of 15598 particles were selected from 1650 micrographs. An initial model was generated by filtering the crystal structure of CV-A16 (PDB: 5C4W)^5^ to 50 Å resolution with Relion 1.3^38^. 3D classification of the particles resulted in essentially five classes of particles: mature viruses (~25%), virus–receptor complexes (~10%), A-particles (~10%), virus–receptor complexes with lower receptor occupancy and the other irregular particles. All refinement and post processing was performed with Relion 1.3. The final density maps were calculated using 3900 particles for the mature viron, 1597 particles for CV-A10/KRM1 complex and 1578 particles for the A-particle, with an overall resolution of 3.5 Å, 3.9 Å and 4.4 Å, respectively, estimated by Fourier shell correlation^38^ (Supplementary Fig. 3a). Particles from the other irregular class were discarded. We then used localized extraction to extract virus bound receptors^39^. 26812 receptors could be extracted from 1597 particles, which gave an approximate occupancy of 28%.

### Model building and refinement

The EM structure of a mature CV-A10 virus (isolated from Zhejiang, China; PDB: 6AKS^14^), which has 94% sequence identity to our virus strain, was fitted into the electron potential map of our mature CV-A10 and rebuilt with COOT^40^. The model was improved using Phenix.real_space_refine^41^. The refined structure of the mature virion and the crystal structure of KRM1 (PDB: 5FWT)^21^ were then fitted into the density map of the CV-A10/KRM1 complex. The structure of the CV-A10 A-particle was built based on the structure of an A-particle of CV-A10 Zhejiang isolate (PDB: 6AKT)^14^. Both structures of the receptor complex and A-particle were also subjected to real space refinement with Phenix with tight stereochemistry restraints. Refinement statistics are given in Supplementary Table 1.

### Plate-based thermal-shift assay (PaSTRy)

SYTO9 dye (Invitrogen) was used as fluorescent probe to detect the RNA exposure^42^. Reactions of total volume 50 µl were set up in a semi-skirted 96-well PCR plate (4 Titude, Surrey, U.K.), containing 0.4 μg of the CV-A10, 5 μM SYTO9 and the KRM1/DKK1 complex at different concentrations (from 0.35 to 10 μg/well), or control protein (BSA) or just the buffer (50 mM Hepes pH7.4, 200 mM NaCl). The plate was heated in an Mx3005p qPCR machine (Agilent Technologies, USA) from 24 to 98 °C at a rate of 1 °C min^–1^. Fluorescence changes were monitored at 1 °C intervals with excitation and emission wavelengths at 492 nm and 585 nm, respectively. The experiment was done in triplicate.

### **Figures**

Residues forming the virus–receptor interface were identified with PISA^43^. Roadmaps were produced using Rivem^44^. All other figures were prepared with PYMOL^45^ and CHIMERA^46^.

### Reporting summary

Further information on research design is available in the Nature Research Reporting Summary linked to this article.

## Supplementary information

Supplementary Information

Reporting Summary

## Data Availability

The coordinates of CV-A10 mature viron, CV-A10/KRM1 complex and CV-A10 A-particle are available from the PDB with accession codes 6SMG, 6SNW and 6SNB, respectively. The corresponding Cryo-EM density maps are available from EMDB, accession codes EMD-10242, EMD-10263 and EMD-10256. The data that support the findings of this study are available from the corresponding authors on request.
