# Supplementary material for: Hand-foot-and-mouth disease virus receptor KREMEN1 binds the canyon of Coxsackie Virus A10
Source: Nat Commun. 2020 Jan 7;11:38. doi: 10.1038/s41467-019-13936-2 (PMC6946704; doi:10.1038/s41467-019-13936-2)
Supplement: Supplementary file 1 — Supplementary Information [file 41467_2019_13936_MOESM1_ESM.pdf]

Supplementary Information

Hand-foot-and-mouth disease virus receptor KREMEN1 binds the canyon of Coxsackie Virus A10

Zhao et al.

This file contains Supplementary Figures 1-12 and Supplementary Tables 1 and 2.

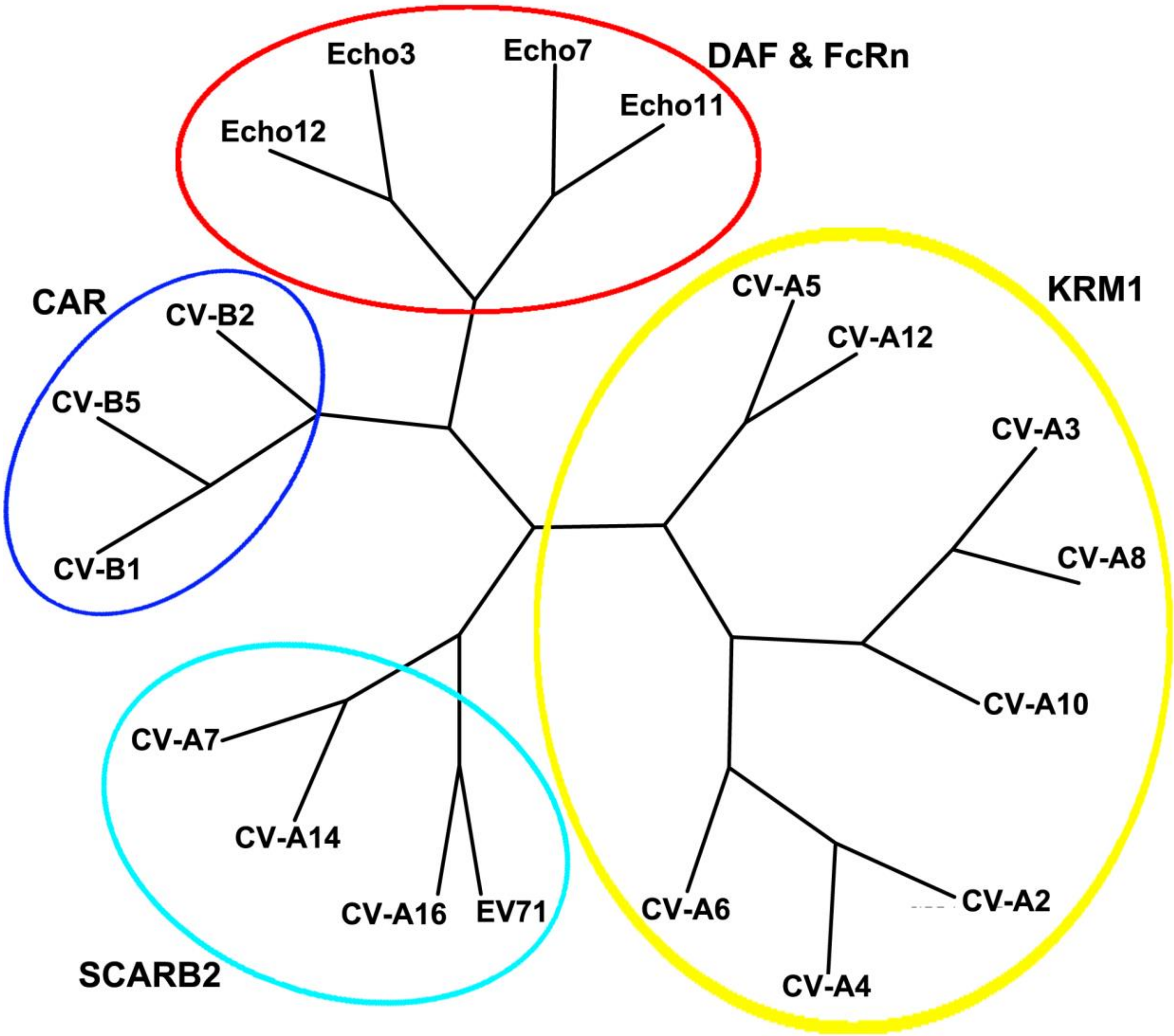

Supplementary Figure 1 | Unrooted phylogenetic tree of some HFMD-causing enteroviruses derived by comparing the capsid sequences. Viruses using KRM1, SCARB2, CAR and DAF/FcRn as receptors are circled in yellow, cyan, blue and red, respectively.

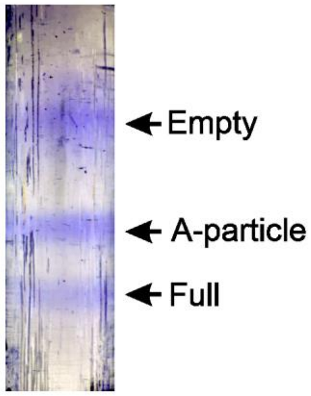

Supplementary Figure 2 | Zonal ultracentrifugation. Purification of CV-A10 sample using 15 - 45% sucrose gradient at 105,000 g for 3 h at 4 °C resulted in 3 bands in positions typically seen for enreroviruses[1]. The top band corresponds to empty particle containing no RNA. The middle band corresponds to A-particles containg RNA. The bottom band is composed of mature viruses.

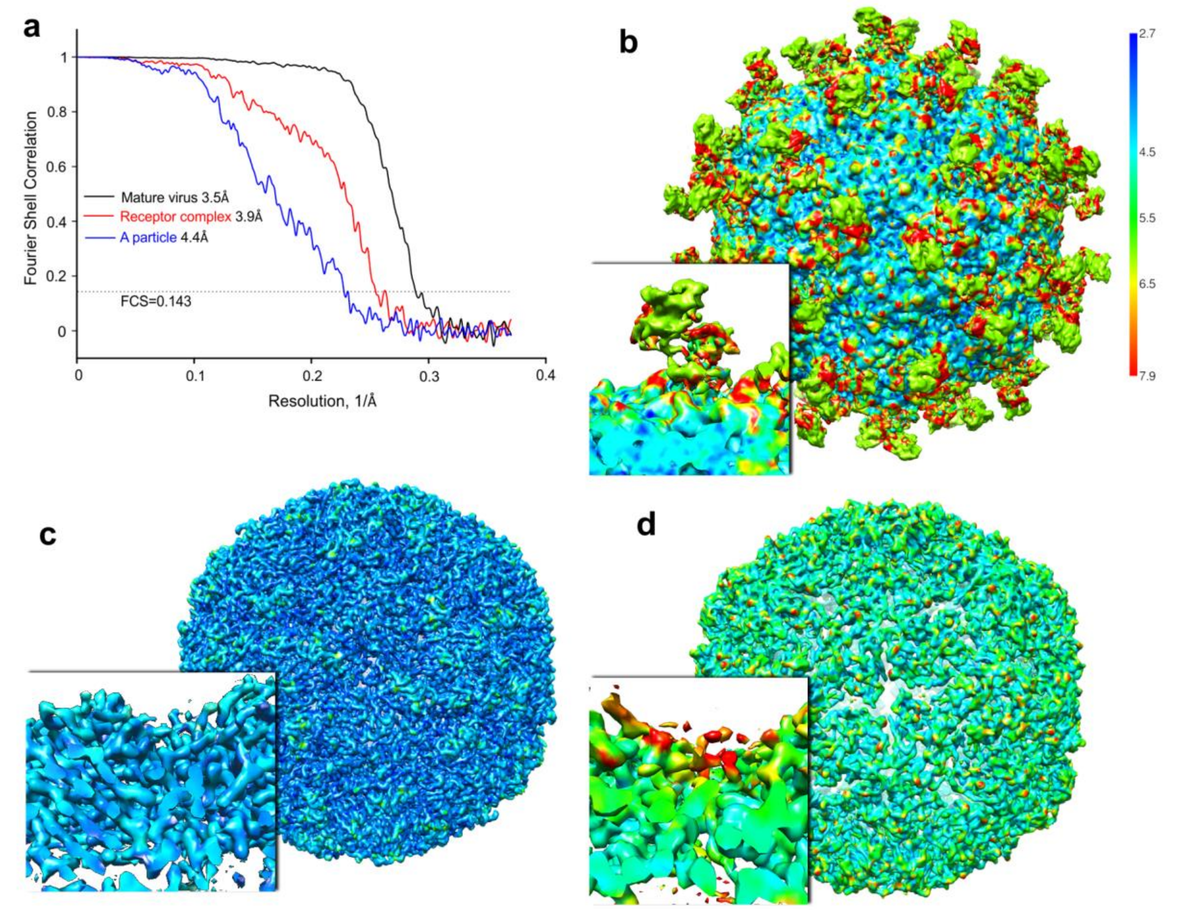

Supplementary Figure 3 | Overall quality of EM maps. a, The gold standard Fourier shell correlation curves of the final maps with resolutions (for FSC cut-off of 0.143) of 3.5 Å for CV-A10 mature virus (black line), 3.9 Å for CV-A10/KRM1 complex (red line) and 4.4 Å for the CV-A10 A-particle (blue line); **b**-**d**, the final maps coloured by local resolution as calculated by Xmipp MonoRes for: CV-A10/KRM1 complex (**b**), mature virus (**c**) and expanded A-particle (**d**). Close-ups of the central section for each map are shown as insertions in panels **b** to **d**. The resolution scale bar shown to the right of **b** applies to all maps.

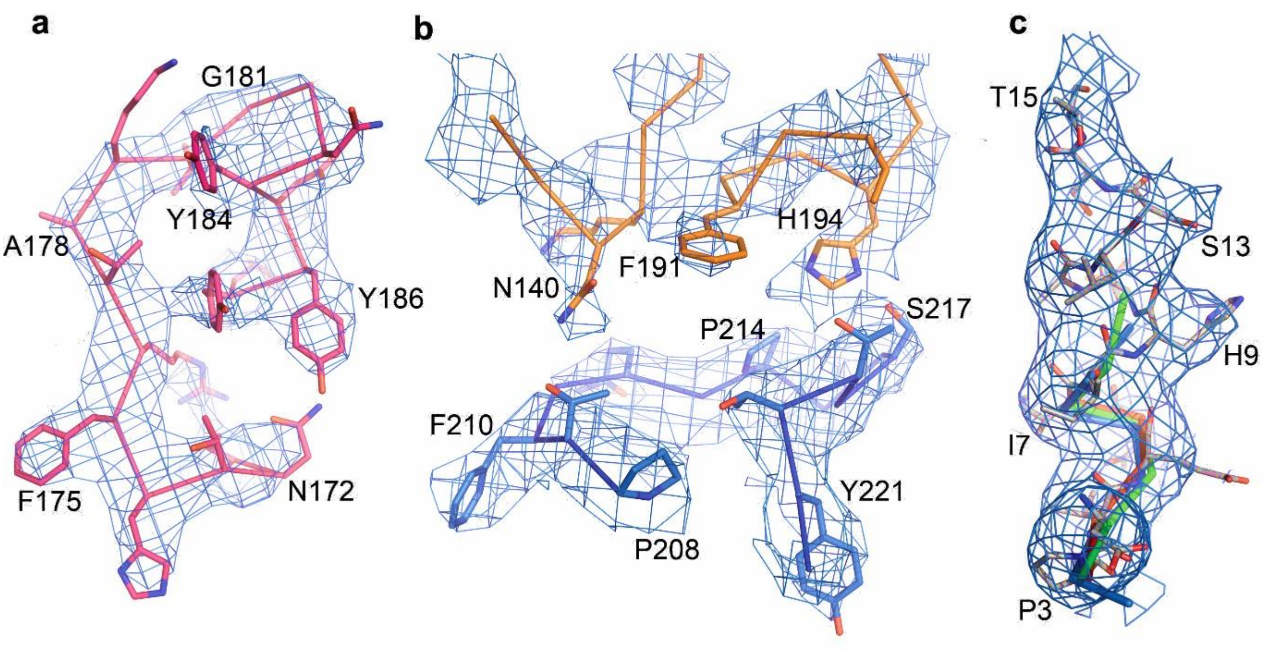

Supplementary Figure 4 | Density maps. a, Density (blue mesh) for the CV-A10 VP3 GH-loop (red sticks) in the CV-A10/KRM1 complex. **b**, Density at the CV-A10 (blue sticks)/KRM1 (orange sticks) interface. **c**, Density for the VP1 N-terminal helix of the CV-A10 mature viron (grey sticks) in which more residues are ordered than in the CV-A10 structures of three other genotypes deposited in the Protein Data Bank (blue (PDB:6AKS), green (PDB:6ACU) and orange (PDB:6IIJ) sticks).

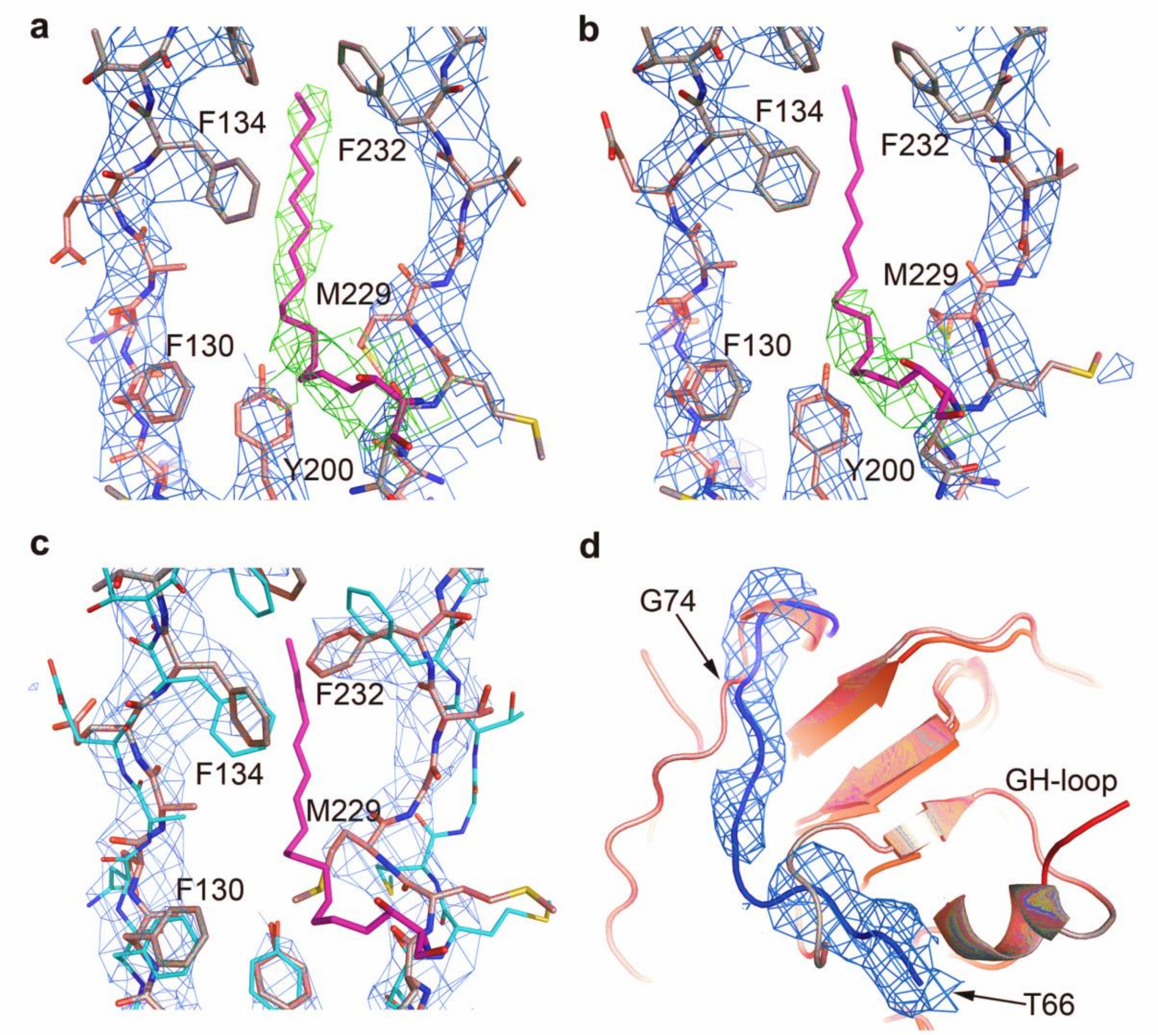

Supplementary Figure 5 | Density maps showing structural differences between the mature virion, receptor bound complex and A-particle. a, b, Density in the VP1 pocket region of the mature virus (**a**) and the CV-A10/KRM1 complex (**b**), protein drawn as grey sticks and pocket factor (pocket factor, modelled as sphingosine) as magenta sticks. The contour level for the pocket factor (green mesh) is 50% of that for the capsid (blue mesh), suggesting lower occupancy. **c**, Density at the VP1 pocket factor binding region of the A-particle. A-particle residues drawn as grey sticks and the corresponding residues and bound pocket factor of the mature virion as cyan and magenta sticks, showing the collapsed pocket in the A-particle. **d**, Density for residues 66-70 of VP1 of the A-particle showing the N-terminus of VP1 (blue) externalising through an opening adjacent to the VP3 GH-loop (VP3 is shown in red). The mature virus is shown in grey for comparison. The interior is at the top in this panel.

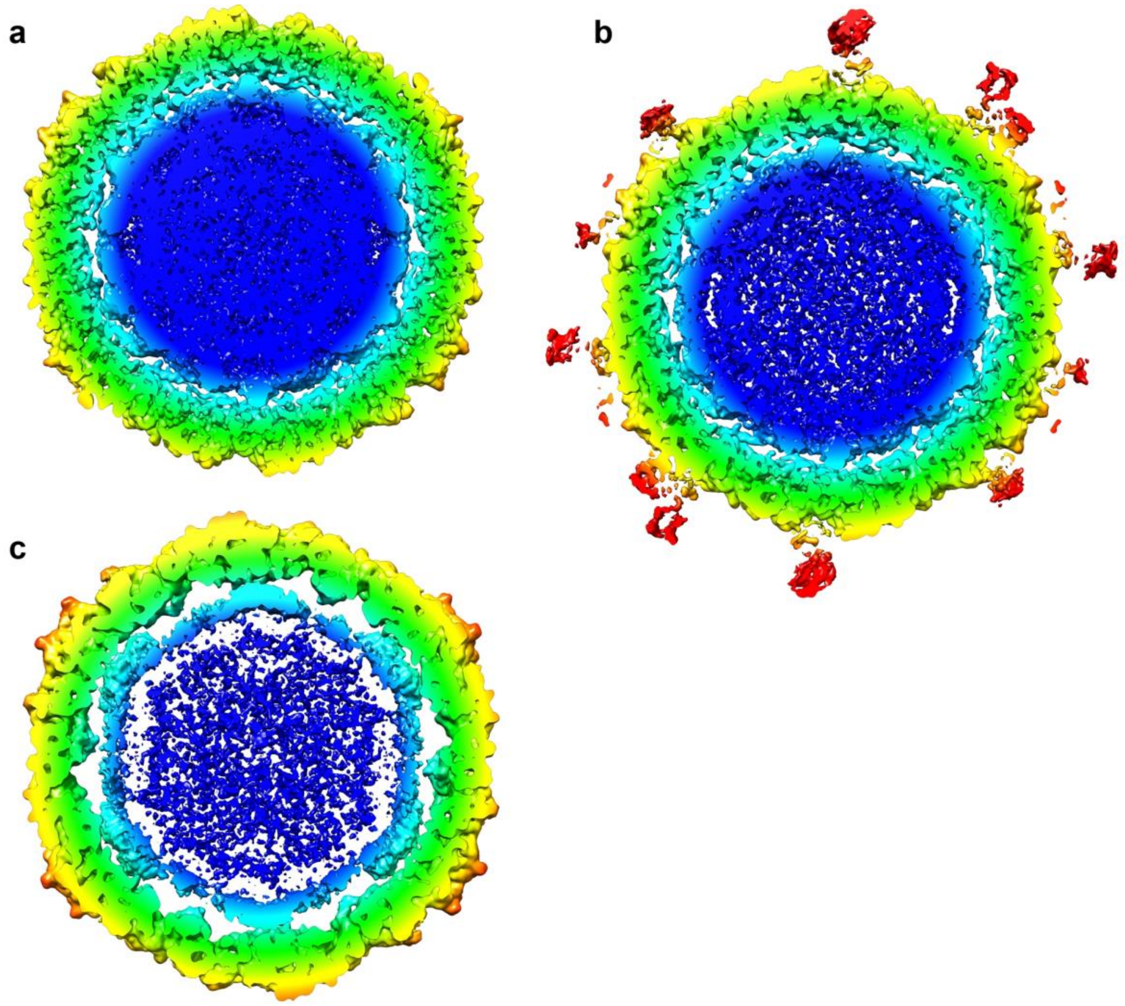

Supplementary Figure 6 | RNA structure inside the virus capsid. a-c Density maps coloured by radius from blue to red showing the layered RNA structure within the capsid of mature virus (**a**), receptor complex (**b**) and the A-particle (**c**). The gap between capsid and RNA in **c** is due to the particle expansion and the externalisation of VP4 and the N-terminus of VP1.

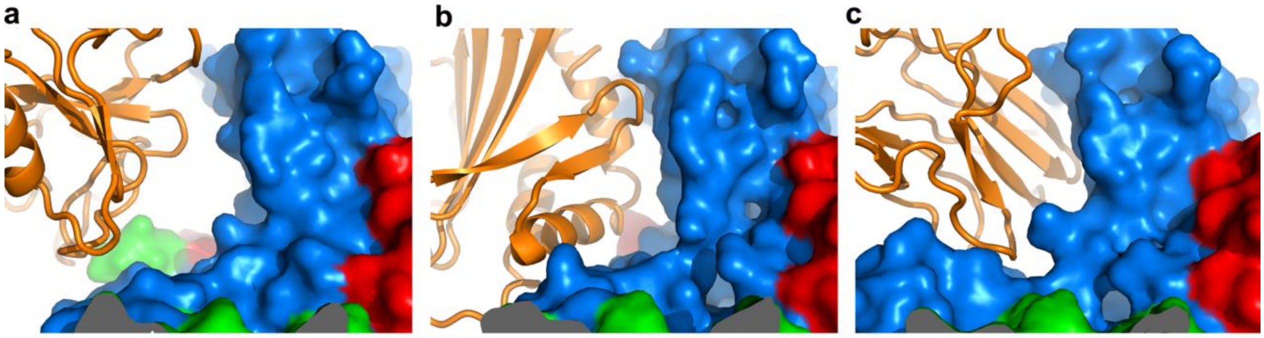

Supplementary Figure 7 | KRM1 spans the width of the canyon but does not reach the canyon floor. Side views of the canyon depression for CV-A10/KRM1 complex (**a**), E6/FcRn complex (**b**) and PV1/CD155 complex (**c**). The viruses are drawn as a surface with VP1 in blue, VP2 in green and VP3 in red. The receptors are drawn as cartoons in orange.

a

```
                 VP1
                     * *            *    * ** **              *           ** *   * *
AJK93556         159-APKPTGR 206-GYPTFGQHPETSNTTYGLCPN 237-VSREA 286-SARDRSSIKQANM
A0A0C5AWF6*          APKPTGR     GYPTFGQHPETSNTTYGLCPN     VSREA     SARDRSSIKQANM
ANZ90273             APKPTGR     GYPTFGQHPETSNTTYGLCPN     VSREA     SARDRSSIKQANM
ANT96619             APKPTGR     GYPTFGQHPETSNTTYGLCPN     VSREA     SARDRSSIKQANM
ALB72972             APKPTGR     GYPTFGQHPETSNTTYGLCPN     VSREA     SARDRSSIKQANM
AQZ26616             APKPTGR     GYPTFGQHPETSNTTYGLCPN     VSREA     SARDRSSIKQANM
ANZ90275             APKPTGR     GYPTFGQHPETSNTTYGLCPN     VSREA     SARDRSSIKQANM
AQZ26615             APKPTGR     GYPTFGQHPETSNTTYGLCPN     VSREA     SARDRSSIKQANM
AWU78864             APKPTGR     GYPTFGQHPETSNTTYGLCPN     VSREA     SARDRSSIKQANM
A0A1V0FT21*          APKPTGR     GYPTFGQHPETSNTTYGLCPN     VSREA     SARDRSSIKQANM
ALB72969             APKPTGR     GYPTFGQHPETSNTAYGLCPN     VSREA     SARDRSSIKQANM
A0A1B3Z4Y8*          APKPTGR     GYPTFGQHPETSNTTYGLCPN     VSREA     SARDRSSIKQANM
ALB72961             APKPTGR     GYPTFGQHPETSNTTYGLCPN     VSREA     SARDRSSIKQANM
AY421767*            APKPTGR     GYPTFGQHPETSNTTYGQCPN     VSRVA     SARDRASIKQANM

                 VP2                   VP3
                        ** ****          *           * *****               ***    *
AJK93556         133-AGRGSNTKPNEAPHP 56-NNTTE 176-RTAKTGGNYDYYTAG 229-DTDEVTQQAVLQ
A0A0C5AWF6*          AGRGSNTKPNEAPHP    NNTTE     RTAKTGGNYDYYTAG     DTDEVTQQAVLQ
ANZ90273             AGRGSNTKPNEAPHP    NNTTE     RTAKTGGNYDYYTAG     DTDEVTQQAVLQ
ANT96619             AGRGSNTKPNEAPHP    NNTTE     RTAKTGGNYDYYTAG     DTDEVTQQAVLQ
ALB72972             AGRGFNTKPDEAPHP    NNTTE     RTAKTGGNYDYYTAG     DTDEVTQQAVLQ
AQZ26616             AGRGSNTKPNEAPHP    NNTTE     RTAKTGGNYDYYTAG     DTDEVTQQAVLQ
ANZ90275             AGRGSNTKPNEAPHP    NNTTE     RTAKTGGNYDYYTAG     DTDEVTQQAVLQ
AQZ26615             AGRGSNTKPNEAPHP    NNTTE     RTAKTGGNYDYYTAG     DTDEVTQQAVLQ
AWU78864             AGRGSNTKPNEAPHP    NNTTE     RTAKTGGNYDYYTAG     DTDEVTQQAVLQ
A0A1V0FT21*          AGRGSNTKPNEAPHP    NNTTE     RTAKTGGNYDYYTAG     DTDEVTQQAVLQ
ALB72969             AGRGSNTKPNEAPHP    NNTTE     RTAKTGGNYDYYTAG     DTDEVTQQAVLQ
A0A1B3Z4Y8*          AGRGSNTKPNEAPHP    NNTTE     RTAKTGGNYDYYTAG     DTDEVTQQAVLQ
ALB72961             AGRGSNTKPNEAPHP    NNTTE     RTAKTGGNYDYYTAG     DTDEVTQQAVLQ
AY421767*            AGRGSNTKPNEAPHP    NNTTD     RTAKTGGNYDYYTAG     DTDEVTQQAVLQ
```

b

```
            VP1
                 * *            *    * ** **              *           ** *   * *
CV-A10      159-APKPTGR 206-GYPTFGQHPETSNTTYGQCPN 237-VSRVA 286-SARDRASIKQANM
CV-A2           APKPDGR     GYPTFGEHSGEDSLRYGQCPN     VSEEI     TANRPTITNT---
CV-A4           APKPDAR     GYPTFGPHSETSNLSYGQCPN     VSKQI     AKDRRKITET---
CV-A6           APKPDGR     GYPTFGEHKQATNLQYGQCPN     VSEST     AADRASITTT---
CV-A3           APKPQER     GYPTFDDRPQTSNRPYGQCPN     VSKTP     SAKDREDIKNT--
CV-A8           APKPQER     GYPTFDDRPATSNHPYGQCPN     VSKTP     TSKDRQSIKTT--
CV-A5           APVPTGR     GYPTFGEVPVTTNLNYGQCPN     VSGVS     ASSNRTSITTT--
CV-A12          APVPTGR     GYPTFGERPVTTNMNYGQCPN     VSGEA     ASHNRASITSNT-

            VP2                     VP3
                    ** *** *           *           * *****               ***    *
CV-A10      133-AGRGSNTKPN-EAPHP 56-NNTTD 176-RTAKTGGNYDYYTAG 229-DTDEVTQQAVLQ
CV-A2           AEQDATQKPN-TAKHP    NNTSK     RAVETGGVFDYYTTG     DTDEITQQATLQ
CV-A4           ASKSERSKPNDPAQYP    NNVTD     RTVKTGGYLDYYATG     DTPEITQQAVLQ
CV-A6           AASSPATKPNGQGLYP    NNTTG     RAVKTGGVYDYYATG     DTDEIRQTAEYQ
CV-A3           AAASPEKKPT-TAPHP    NNVTG     RTGKTGGYYDYYASG     DTDEVTQQAILQ
CV-A8           GANSSDPKPN-TAKHP    NNVTG     RTGKVGGYFDYYASG     DTDEVTQQAVLQ
CV-A5           AGMGAGDKPS-TAPHP    NNTTD     RTVETGGILDYYSTG     DTESISQTAILQ
CV-A12          AGMGPGDKPQ-NAPHP    NNVTN     RTVETGGILDYYSTG     DTESISQTAILQ
```

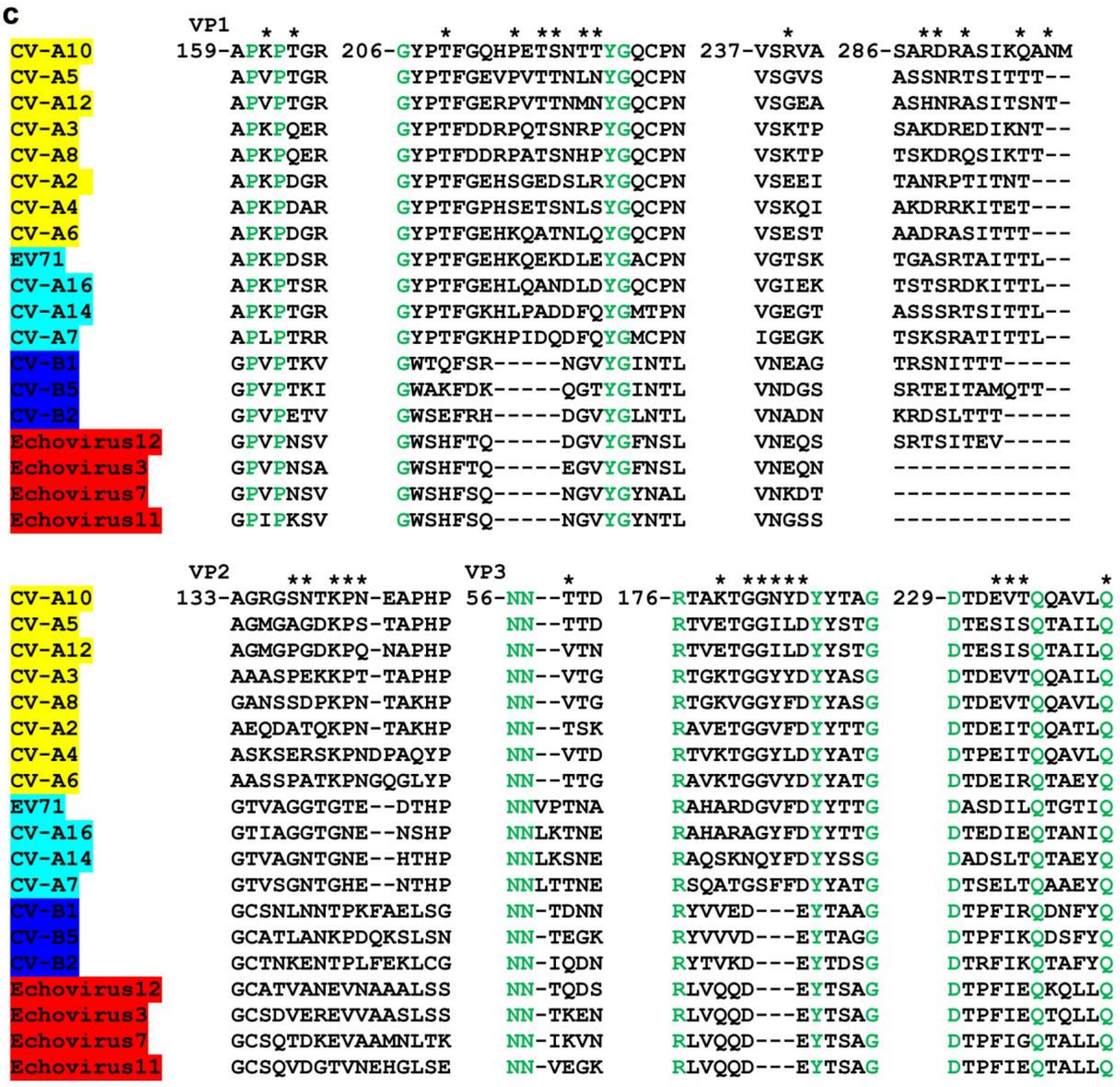

Supplementary Figure 8 | Comparison of residues in the receptor attachment area of CV-A10 with other HFMD causing enteroviruses. a, Sequence alignment of the receptor binding region of 14 CV-A10 strains, including the 4 structurally known strains (marked with *). **b**, Sequence alignment of the receptor binding region of CV-A10 with the HFMD causing type A enteroviruses that use KRM1 as cell entry receptor. **c**, Sequence alignment of the receptor binding region of CV-A10 with the HFMD causing enteroviruses. Virus names with yellow, cyan, blue and red background are KRM1, SCARB2, CAR and DAF dependent, respectively. In all three panels, conserved residues are in green; residues of CV-A10 that have close contacts with KRM1 (≤ 4Å) are marked with *s on top.

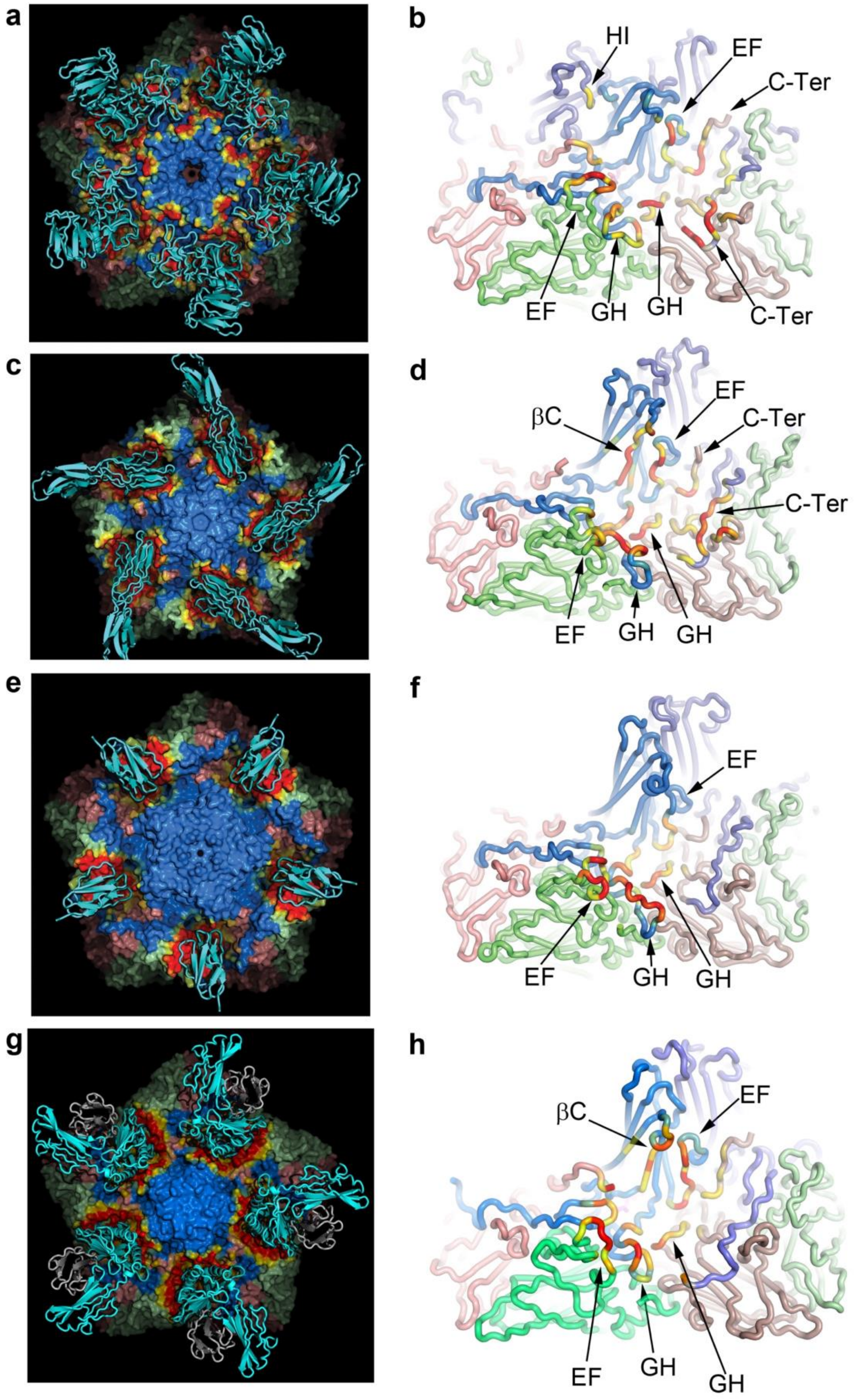
a
b
HI
EF
C-Ter
EF
GH
GH
C-Ter
c
d
βC
EF
C-Ter
C-Ter
EF
GH
GH
e
f
EF
EF
GH
GH
g
h
βC
EF
EF
GH
GH

Supplementary Figure 9 | Comparison of attachment of canyon binding receptors to CV-A10, PV-1, CV-A24v and E6. a. A pentameric unit of CV-A10 shown as surface representation with the bound receptor shown as cyan ribbons. VP1, VP2 and VP3 of CV-A10 are in blue, pale green and salmon, respectively. Virus atoms with distances ≤ 4.0 Å to the receptor are coloured in red, distances > 4.0 Å and ≤ 9.0 Å in yellow. **b**, Receptor attachment area of CV-A10. The backbones of the capsid protein are shown as thick worms with VP1, VP2 and VP3 of the central protomer coloured in blue, green and salmon respectively, the neighbouring protomers are in pale colours. The colour scheme for residues involved in receptor interactions is as in **a**. **c-d, e-f and g-h,** Figures corresponding to **a** and **b** showing virus-receptor interactions for PV-1/CD155 (PDB ID, 3J8F), CV-A24v/ICAM-1 (PDB ID, 6EIT) and echovirus 6/FcRn (PDB ID, 6ILM), respectively. In CV-A10/KRM1 complex, residues from three biological protomers are involved in receptor binding, whereas residues from two biological protomers are involved in PV-1/CD155 (**c**, **d**), CV-A24v/ICAM-1 (**e**,**f**) and echovirus 6/FcRn (**g**, **h**) complexes.

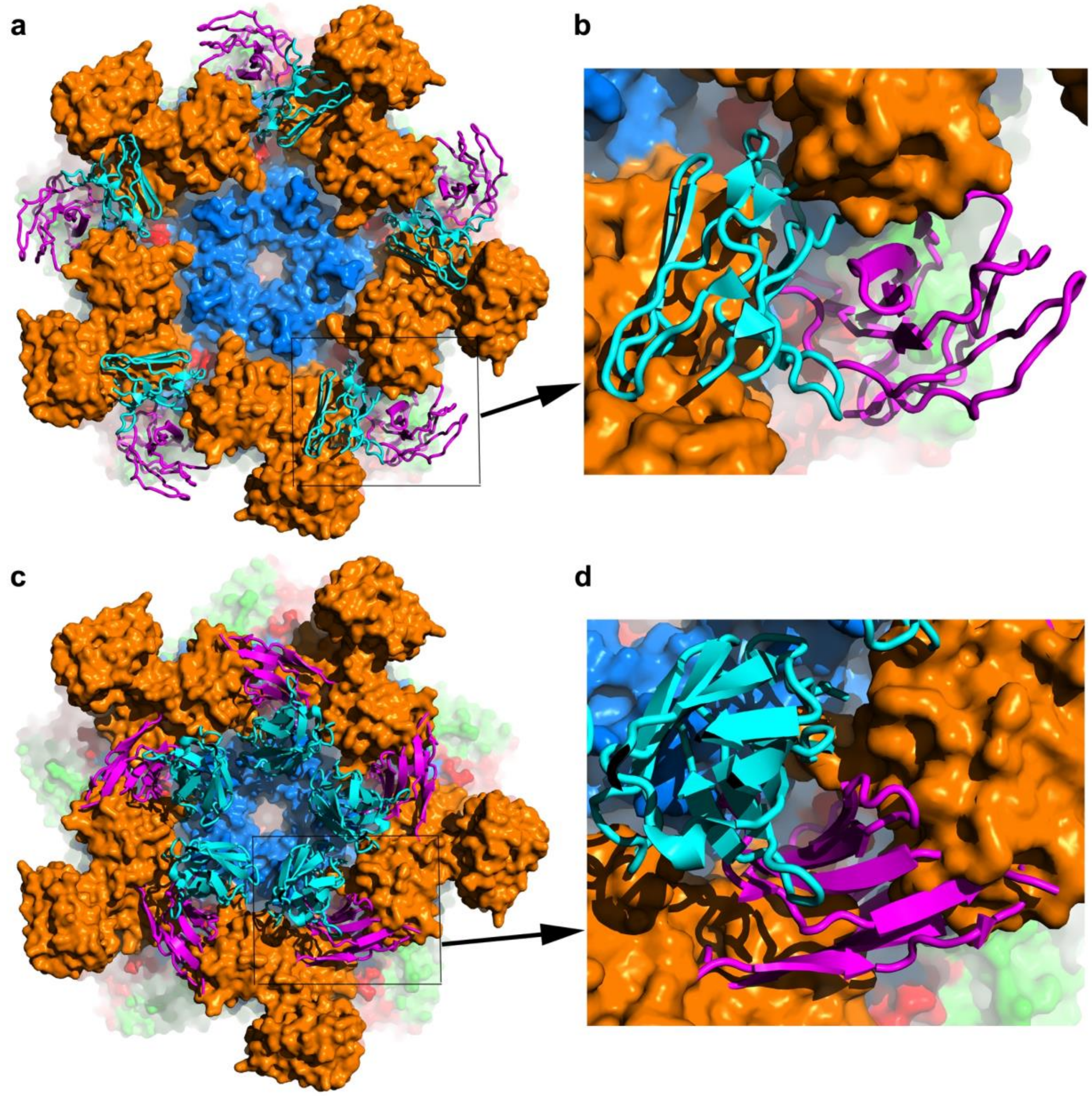

Supplementary Figure 10 | Comparison of engagement modes of KRM1 with anti-CV-A10 2g8 and anti-CV-A6 1D5. a, Superimposition of a CV-A10/KRM1 pentamer with that of CV-A10/2g8-Fab. CV-A10 and KRM1 are shown as surface representations with VP1, VP2, VP3 and KRM1 in blue, green, red and orange respectively. Only the variable domains of 2g8 are modelled in the CV-A10/2g8 complex and shown here as ribbons with Vh in magenta and Vl in cyan. **b,** Closeup showing severe clashes of 2g8 with KRM1. **c**, Binding position of the variable domains of the CV-A6 antibody 1D5 relative to the position of KRM1 on CV-A10. **d**, Closeup showing the clashes between the antibody and the receptor. The colour scheme in **c** and **d** is the same as in **a** and **b**.

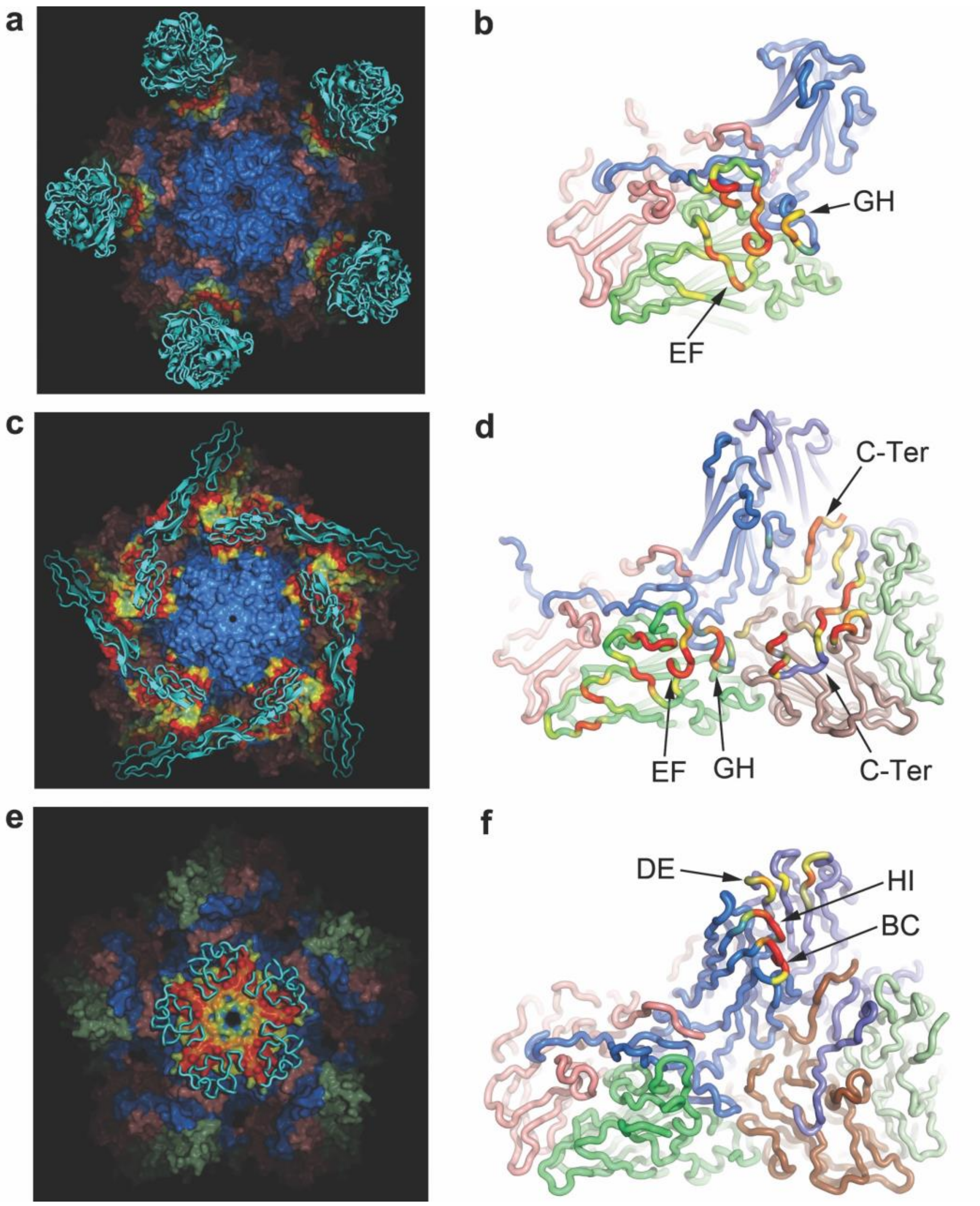

Supplementary Figure 11 | Comparison of non-canyon binding receptor attachment to EV-A71, E6 and HRV2. a, A pentameric unit of EV-A71 shown as surface representation with the bound receptor shown as cyan ribbons. VP1, VP2 and VP3 of CV-A10 are in blue, pale green and salmon, respectively. Virus atoms with distances ≤ 4.0 Å to the receptor are coloured in red, distances > 4.0 Å and ≤ 9.0 Å in yellow. **b**, Receptor attachment area of EV-A71. The backbones of the capsid protein are shown as thick worms with VP1, VP2 and VP3 coloured in blue, green and salmon respectively. The colour scheme for residues involved in receptor interactions is as in **a**. **c-d,** Interactions of E6 and its attachment receptor DAF (PDB ID, 6ILK). **e-f**, Interactions of HRV2 and its attachment receptor LDLR (PDB ID, 3DPR). The drawing style and colour scheme of **c-d** and **e-f** are as in **a** and **b**.

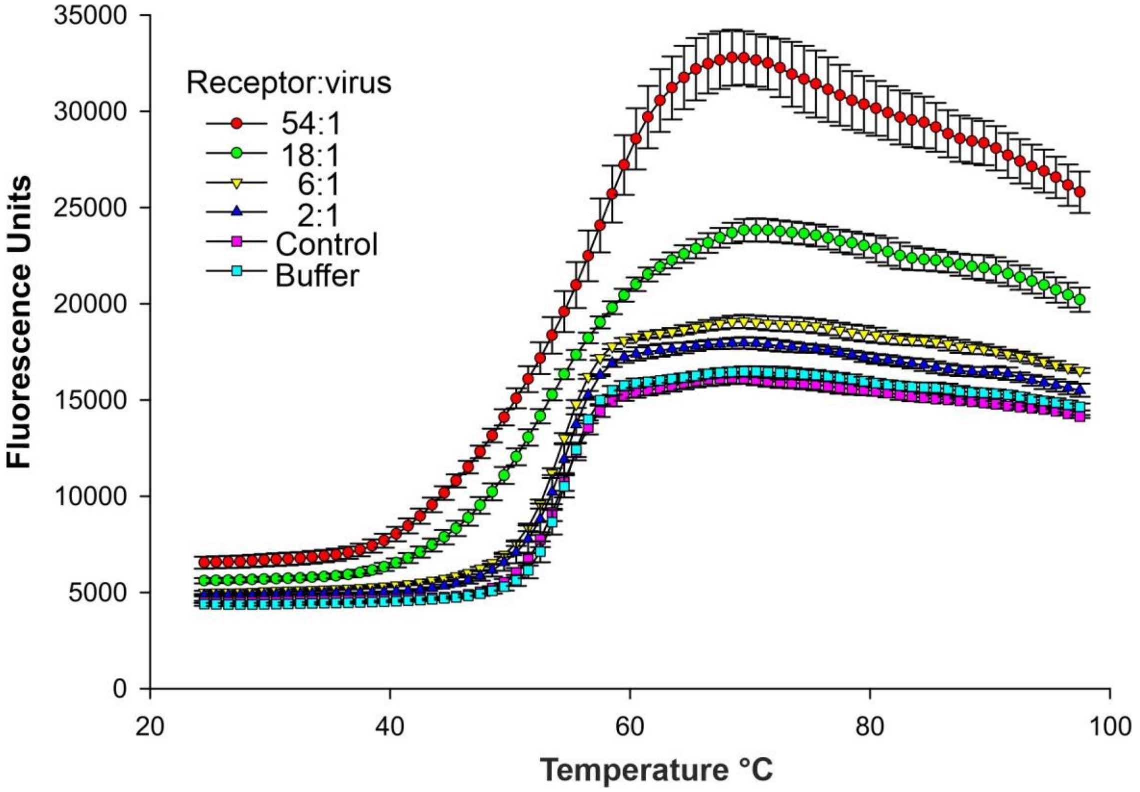

Supplementary Figure 12 | KRM1 destabilizes CV-A10 detected by a plate-based thermal-shift assay (PaSTRy). The differential scanning fluorimetry assays were performed with the virus and the receptor mixed together with the dyes SYTO9 for detection of RNA exposure. The raw fluorescence traces are shown as different coloured lines with error bars indicating the viral RNA was released earlier in the receptor concentration dependant manner. Tm is the inflection point on the curve – Virus: 55 °C; highest receptor concentration: 49 °C; second highest: 53 °C; third highest: 54 °C; others show no change.

Supplementary Table 1 | Cryo-EM data collection, reconstruction and refinement statistics

	Mature viron	CV-A10/KRM1	A-particle
**Data collection and reconstruction**			
Voltage (kV)	300		
Frames	32		
Dose rate (e^-/Å^2/s)	4		
Total dose (e^-/Å^2)	35		
Pixel size (Å)	1.35		
Defocus (µm)	0.5 - 2.5		
Movies	1650		
Particles used in the final reconstruction	3900	1597	1578
Map resolution (Å)	3.5	3.9	4.4
Map sharpening B-factor (Å^2)	-132	-143	-131
**Model refinement**			
Total number of atoms	6411	8744	5382
Protein residues	829	1122	708
Model-to-map fit, CC_mask	85.9	81.7	84.3
R.m.s.d., bonds (Å)	0.006	0.007	0.006
R.m.s.d., angles (°)	0.6	0.7	0.7
All-atom Clash score	9.9	10.3	13.7
Rotamer outliers (%)	0	0	0.2
Ramachandran plot			
Favored (%)	92.9	87.8	88.0
Allowed (%)	7.1	12.2	12.0
Outliers (%)	0	0	0

Supplementary Table 2 | Comparison of enterovirus-receptor interface.

Virus/receptor	**CV-A10 /KRM1**	**PV-1 /CD155**	**CV-A24 /ICAM**	**EV71 /SCARB2**	**Echovirus 6 /DAF**	**Echovirus 6 /FcRn**
No. of virus residues at interface	52	58	38	27	70	52
Interface area ($Å^2$)	1370	1520	1220	726	1900	1400
No. of potential H-bonds	10	17	16	5	21	15
No. of potential salt bridges	4	4	11	2	0	2

References

1 Ren, J. *et al.* Picornavirus uncoating intermediate captured in atomic detail. *Nat Commun* **4**, 1929, doi:10.1038/ncomms2889 (2013).
